# Supplementary material for: Effect of Imipenem and Amikacin Combination against Multi-Drug Resistant Pseudomonas aeruginosa
Source: Antibiotics (Basel). 2021 Nov 22;10(11):1429. doi: 10.3390/antibiotics10111429 (PMC8615098; doi:10.3390/antibiotics10111429)
Supplement: Supplementary file 1 [file antibiotics-10-01429-s001.zip › antibiotics-1451998-supplementary.pdf]

Supplementary Data

Table S1: Distribution of *bla_{IMP}* genotype among 45 isolated *P. aeruginosa*

Infections	No.of isolates	*bla_{IMP}* positive isolates No. (%)*,**
Wound	17	6 (37.5%)
Ear infections	6	4 (25%)
Burn infections	5	3 (18.75%)
Chest infections	8	3 (18.75%)
Urinary tract infections	5	0
Gastroenteritis infections	4	0
Total	45	16 (35.6%)**

*Percent correlated to total no. of positive isolates, ** percent correlated to total no of *P. aeruginosa* isolates.

Table S2: Distribution of *aaac(6′)-Ib* genotype among 45 isolated *P. aeruginosa*

Infections	No.of isolates	*aaac(6′)-Ib* positive isolates No. (%)*,**
Wound	17	8 (42.1%)
Ear infections	6	2 (10.5%)
Burn infections	5	2 (10.5%)
Chest infections	8	3 (15.8%)
Urinary tract infections	5	2 (10.5%)
Gastroenteritis infections	4	2 (10.5%)
Total	45	19 (42.2%)**

*Percent correlated to total no. of positive isolates, ** percent correlated to total no of *P. aeruginosa* isolates.

Table S3: Comparisons between different groups of *Pseudomonas aeruginosa* resistant to both Imipenem & Amikacin in different times

At 0.5x MIC

	Control group (I)	Amikacin group (II)	Imipenem group (III)	Combination group (IV)	P value
0 h	8.21±0.02	8.21±0.02	8.21±0.02	8.21±0.02	*1*
2 h	9.41±0.01	9.42±0.03	9.40±0.01	9.41±0.01	*0.045**
4 h	11.96±0.01	11.95±0.02	11.96±0.02	9.30±0.02	*<0.001**
8 h	10.20±0.02	10.20±0.01	10.20±0.01	9.21±0.01	*<0.001**
12 h	10.42±0	10.42±0.01	10.42±0.01	8.96±0.05	*<0.001**
24 h	10.47±0	10.47±0	10.47±0.01	7.48±0	*<0.001**

At 1 x MIC

	Control group (I)	Amikacin group (II)	Imipenem group (III)	Combination group (IV)	P value
0 h	8.21±0.02	8.21±0.02	8.21±0.02	8.21±0.02	*1*
2 h	9.41±0.01	9.32±0.01	9.35±0.01	9.33±0.02	*<0.001**
4 h	11.96±0.01	9.19±0.03	9.21±0.03	9.05±0.03	*<0.001**
8 h	10.20±0.02	8.03±0.02	8.09±0.04	7.95±0.03	*<0.001**
12 h	10.42±0	7.95±0.03	7.90±0.05	6.84±0.06	*<0.001**
24 h	10.47±0	7±0.04	7.1±0.04	5 ±0.05	*<0.001**

At 2 x MIC

	Control group (I)	Amikacin group (II)	Imipenem group (III)	Combination group (IV)	P value
0 h	8.21±0.02	8.21±0.02	8.21±0.02	8.21±0.02	*1*
2 h	9.41±0.01	9.07±0.02	9.08±0.02	8.02±0.02	*<0.001**
4 h	11.96±0.01	8.91±0.02	8.96±0.02	7.80±0.02	*<0.001**
8 h	10.20±0.02	7.84±0.03	7.82±0.02	6.71±0.07	*<0.001**
12 h	10.42±0	6.88±0.04	6.85±0.02	5.71±0.05	*<0.001**
24 h	10.47±0	6.78±0.04	6.68±0.06	4.59±0.08	*<0.001**

At 4 x MIC

	Control group (I)	Amikacin group (II)	Imipenem group (III)	Combination group (IV)	P value
0 h	8.21±0.02	8.21±0.02	8.21±0.02	8.21±0.02	*1*
2 h	9.41±0.01	7.89±0.02	7.75±0.06	5.72±0.03	*<0.001**
4 h	11.96±0.01	7.58±0.08	7.71±0.03	5.68±0.05	*<0.001**
8 h	10.20±0.02	7.38±0.38	6.68±0.04	4.51±0.03	*<0.001**
12 h	10.42±0	5.60±0.07	5.59±0.07	0±0	*<0.001**
24 h	10.47±0	0±0	0±0	0±0	*<0.001**

Table S4: Comparisons between different groups of *Pseudomonas aeruginosa* resistant to Imipenem only in different times

At 0.5 x MIC

	**Control group (I)**	**Amikacin group (II)**	**Imipenem group (III)**	**Combination group (IV)**	**P value**
0 h	8.21±0.02	8.21±0.02	8.21±0.02	8.21±0.02	*1*
2 h	9.41±0.01	8.90±0.02	9.09±0.02	9.04±0.02	*<0.001**
4 h	11.96±0.01	8.59±0.08	9.26±0.01	8.97±0.02	*<0.001**
8 h	10.20±0.02	7.57±0.09	9.29±0.03	8.73±0.08	*<0.001**
12 h	10.42±0	6.58±0.04	9.41±0.01	8.19±0.10	*<0.001**
24 h	10.47±0	5.53±0.04	10.02±0.01	7.26±0.11	*<0.001**

At 1 x MIC

	**Control group (I)**	**Amikacin group (II)**	**Imipenem group (III)**	**Combination group (IV)**	**P value**
0 h	8.21±0.02	8.21±0.02	8.21±0.02	8.21±0.02	*1*
2 h	9.41±0.01	5.58±0.09	8.95±0.01	7.91±0.04	*<0.001**
4 h	11.96±0.01	4.51±0.03	8.53±0.08	7.52±0.01	*<0.001**
8 h	10.20±0.02	0±0	7.93±0.02	6.90±0.03	*<0.001**
12 h	10.42±0	0±0	6.92±0.06	5.59±0.06	*<0.001**
24 h	10.47±0	0±0	6.20±0.05	4.17±0.06	*<0.001**

Table S5: Comparisons between different groups of *Pseudomonas aeruginosa* resistant to Amikacin only in different times

At 0.5 x MIC

	**Control group (I)**	**Amikacin group (II)**	**Imipenem group (III)**	**Combination group (IV)**	**P value**
0 h	8.21±0.02	8.21±0.02	8.21±0.02	8.21±0.02	*1*
2 h	9.41±0.01	9.41±0.03	6.92±0.04	9.34±0.01	*<0.001**
4 h	11.96±0.01	11.95±0.04	6.54±0.05	9.28±0.02	*<0.001**
8 h	10.20±0.02	10.19±0.02	5.91±0.05	9.12±0.02	*<0.001**
12 h	10.42±0	10.41±0.01	5.55±0.08	8.89±0.06	*<0.001**
24 h	10.47±0	10.47±0.01	4.55±0.05	7.34±0.04	*<0.001**

At 1xMIC

	Control group (I)	Amikacin group (II)	Imipenem group (III)	Combination group (IV)	P value
0 h	8.21±0.02	8.21±0.02	8.21±0.02	8.21±0.02	*1*
2 h	9.41±0.01	8.97±0.02	5.57±0.02	7.85±0.05	*<0.001**
4 h	11.96±0.01	7.72±0.08	4.64±0.04	7.57±0.06	*<0.001**
8 h	10.20±0.02	6.88±0.06	0±0	6.86±0.04	*<0.001**
12 h	10.42±0	6.58±0.08	0±0	6.60±0.06	*<0.001**
24 h	10.47±0	6.21±0.04	0±0	4.2±0.07	*<0.001**

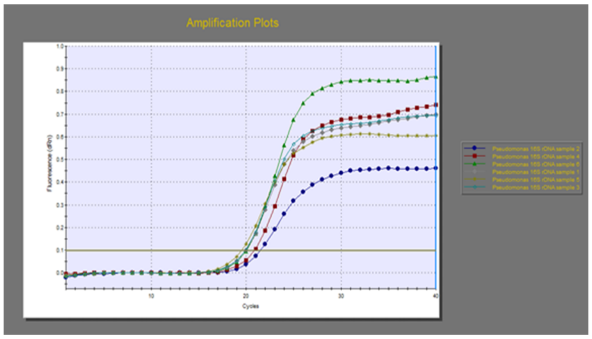

Figure S1 :*P. aeruginosa* 16s rDNA amplification plots

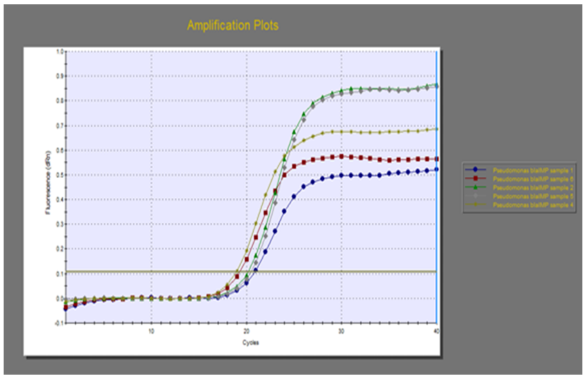

Figure S2:*P. aeruginosa* bla_{IMP} amplification plots

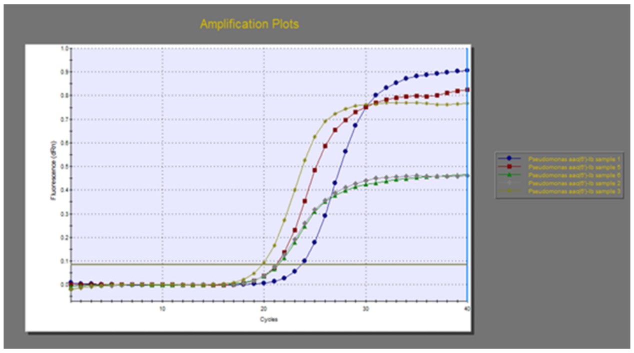

Figure S3 :*P. aeruginosa* bla$_{aac6\text{-}Ib'}$ amplification plots
